# Future projection of greenhouse gas emissions due to permafrost degradation using a simple numerical scheme with a global land surface model

**DOI:** 10.1186/s40645-020-00366-8

**Published:** 2020-10-02

**Authors:** Tokuta Yokohata, Kazuyuki Saito, Akihiko Ito, Hiroshi Ohno, Katsumasa Tanaka, Tomohiro Hajima, Go Iwahana

**Affiliations:** 1 Center for Global Environmental Research, National Institute for Environmental Studies, 16-2 Onogawa, Tsukuba, 305-8506 Japan; 2 Research Center for Environmental Modeling and Application, Japan Agency for Marine-Earth Science and Technology, 3173-25 Showamachi, Kanazawaku, Yokohama, 236-0001 Japan; 3 School of Earth, Energy and Environmental Engineering, Kitami Institute of Technology, 165 Koen-cho, Kitami, 090-8507 Japan; 4 Laboratoire des Sciences du Climat et de l’Environnement (LSCE), Commissariat à l’énergie atomique et aux énergies alternatives (CEA), Gif-sur-Yvette, France; 5 International Arctic Research Center, 739, The University of Alaska Fairbanks, 2160 Koyukuk Dr, Fairbanks, AK 740 99775-7340 USA

**Keywords:** Permafrost degradation, Carbon cycle feedback, Climate change

## Abstract

The Yedoma layer, a permafrost layer containing a massive amount of underground ice in the Arctic regions, is reported to be rapidly thawing. In this study, we develop the Permafrost Degradation and Greenhouse gasses Emission Model (PDGEM), which describes the thawing of the Arctic permafrost including the Yedoma layer due to climate change and the greenhouse gas (GHG) emissions. The PDGEM includes the processes by which high-concentration GHGs (CO_2_ and CH_4_) contained in the pores of the Yedoma layer are released directly by dynamic degradation, as well as the processes by which GHGs are released by the decomposition of organic matter in the Yedoma layer and other permafrost. Our model simulations show that the total GHG emissions from permafrost degradation in the RCP8.5 scenario was estimated to be 31-63 PgC for CO_2_ and 1261-2821 TgCH_4_ for CH_4_ (68^th^ percentile of the perturbed model simulations, corresponding to a global average surface air temperature change of 0.05–0.11 °C), and 14-28 PgC for CO_2_ and 618-1341 TgCH_4_ for CH_4_ (0.03–0.07 °C) in the RCP2.6 scenario. GHG emissions resulting from the dynamic degradation of the Yedoma layer were estimated to be less than 1% of the total emissions from the permafrost in both scenarios, possibly because of the small area ratio of the Yedoma layer. An advantage of PDGEM is that geographical distributions of GHG emissions can be estimated by combining a state-of-the-art land surface model featuring detailed physical processes with a GHG release model using a simple scheme, enabling us to consider a broad range of uncertainty regarding model parameters. In regions with large GHG emissions due to permafrost thawing, it may be possible to help reduce GHG emissions by taking measures such as restraining land development.

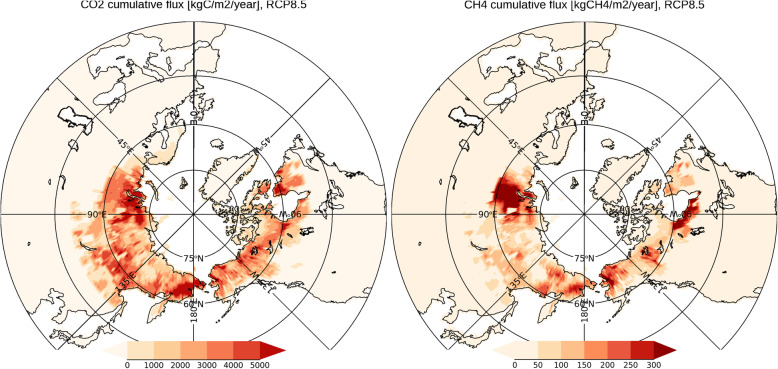

## Introduction

“Permafrost” is the name given to areas where the ground temperature has remained below 0 °C for more than 2 years (IPCC 2013). Virtually all soil contains the bodies of dead organisms (mainly plants) in the form of organic matter (Zimov et al. 2006a; Schuur et al. 2008; Brown 2013). When the soil is not frozen, the organic matter is decomposed by microorganisms and released from the surface to the atmosphere in the form of carbon dioxide or methane (Zimov et al. 2006b; Walter et al. 2007; Ciais et al. 2013). However, when the soil is frozen, the organic matter is trapped without being decomposed, as the activity of these microorganisms is suppressed (Brown 2013; Hugelius et al. 2013; Hugelius et al. 2014). It is estimated that permafrost contains roughly twice the amount of carbon as the air and approximately three times as much as land plants (Prentice et al. 2001; Ping et al. 2008; Tarnocai et al. 2009; Dlugokencky and Tans 2013). As the Earth’s surface temperature rises due to climate change, the frozen soil in the polar region will thaw, thereby releasing in the form of greenhouse gases (GHGs) the organic substances contained in the frozen soil (Collins et al. 2013; Koven et al. 2013; Schuur et al. 2015). These GHGs will further accelerate global warming (Lenton 2012; Köhler et al. 2014; Schuur et al. 2015). Given the large amount of carbon contained in the permafrost, positive feedback from permafrost thawing is very likely to accelerate changes in the climate system (Schaefer et al. 2014; Koven et al. 2015; MacDougall et al. 2015; Schneider von Deimling et al. 2015; MacDougall and Knutti 2016; Steffen et al. 2018; Gasser et al. 2018; McGuire et al. 2018; Kawamiya et al. 2020).

Still, there is a great deal of uncertainty regarding the process of GHG emissions from permafrost thawing (Schaefer et al. 2014). This is partly due to the lack of observational knowledge of basic permafrost processes (Schuur et al. 2015). Although permafrost exists in various forms depending on its formation factors, what has been attracting attention in recent years is the thawing of the Yedoma layer, a permafrost layer containing a large mass of ground ice, mostly found in Alaska and Siberia (Strauss et al. 2013; Strauss et al. 2017). It has long been known that the Yedoma layer exists in permafrost zones (Brouchkov and Fukuda 2002; Schirrmeister et al. 2011; Kanevskiy et al. 2011), but it has only recently been noted that this huge underground layer is thawing rapidly (Vonk et al. 2012; Ulrich et al. 2014; Strauss et al. 2017). Analysis of satellite observations suggest that a subsidence of the ground occurred at sites where tundra fires have caused the heat insulation effect of vegetation on the surface to disappear (Iwahana et al. 2016) and that frozen soil and ground ice are being degraded by erosion from rivers and ocean waves (Günther et al. 2013; Jones et al. 2011; Kanevskiy et al. 2016). Previous studies have reported that the ground ice and frozen soils in the Yedoma layer contain high concentrations of carbon dioxide, methane, and organic carbon (e.g., Saito et al. 2017; Strauss et al. 2017). To date, however, the impact on the climate system of the dynamic degradation of the Yedoma layer associated with ground subsidence has not been sufficiently evaluated, partly due to the difficulty of modeling it in global climate models (Schneider von Deimling et al. 2015).

In this study, we developed a simple scheme to describe the thawing process of the Yedoma layer accompanied by vertical mechanical collapse due to ground subsidence (hereinafter called “dynamic degradation”) based on in-situ observations conducted in Alaska and Siberia. Using this model, we estimate the GHG emissions due to the future degradation of the Yedoma layers. We consider two pathways for GHG emissions due to permafrost degradation: the process of releasing GHGs (CO_2_ and CH_4_) trapped in the frozen soil (referred to as “direct emissions”) and the process of releasing GHG emissions produced by the decomposition of organic matter contained in the frozen soil (“secondary emissions”) caused by the thawing of the permafrost. In addition to the dynamic degradation of the Yedoma layers, we also estimate the GHG emissions due to the thermodynamic degradation of the permafrost owing to the increase in ground temperature. Finally, in the course of our study, we estimate the global mean temperature response caused by the GHG emissions due to permafrost degradations using the simple climate model ACC2 (Tanaka and O’Neill 2018).

## Methods

Permafrost Degradation and Greenhouse gasses Emission Model (PDGEM) evaluates the GHG emissions due to the degradation of the permafrost layer. PDGEM describes the processes of dynamic (Section 2.1.1) and thermodynamic (Section 2.1.2) permafrost degradation with a simple formulation and calculates the GHG emissions globally with a resolution of 1 degree. The parameters used in the formulation are varied (Table 1) in order to describe the future possible behavior of permafrost degradations. Details of the model formulation and experimental settings for the future projections are summarized in the sections that follow.

**Table 1:** Model parameters for the calculation of GHG emissions due to the dynamic and thermodynamic permafrost degradations

Model parameter	Variable	Standard value	Uncertainty range	References
*V*_*dstrb*_ [cm/yr]	Subsidence velocity of burnt area in Yedoma layer	2.4	± 2.1	a
*r*_*ice*_	Ice contents in Yedoma layer	0.64	± 0.15	b
*d*_*soc*_ [m]	Depth of average soil organic layer	3.0	± 1.5	c
π	Fraction for the flux of soil organic carbon, fast pool slow pool	0.025 0.45	0.01–0.04 0.30–0.60	d, e, f, g, h, i
*τ* [year]	Time scale for slow decomposition at 5 °C	25	10-40	d, e, f, h
*∆r*_*wtlnd*, max_ [%]	Maximum increase of wetland fraction	20	10-30	d
Q10	Temperature sensitivity parameter, aerobic anaerobic	2.5 3.0	1.5–3.5 4.0–6.0	d, i, j
*r*_gas_	GHG production ratio, CH_4_:CO_2_ in aerobic CH_4_:CO_2_ in anaerobic Fast pool Slow pool	1:50 1:1 1:7	± 50% ± 20% ± 50%	k, m, n, p
*oxd*	CH_4_ oxidation rate, anaerobic	0.20	0.15–0.40	h

The standard values and uncertainty ranges of the parameters are shown. References for the standard values and uncertainty ranges are as follows. a: Iwahana et al. (2016), b: Strauss et al. (2017), c: Saito et al. (2020), d: Schneider von Deimling et al. (2015), e: Sitch et al. (2003), f: Dutta et al. (2006), g: Koven et al. (2011), h: Burke et al. (2012), i: Schadel et al. (2014), j: Walter and Heimann (2000), k: Lee et al. (2012), m: Schuur et al. (2008), n: Segers (1998), p: Walter Anthony et al. (2014)

### Description of Permafrost Degradation and GHG Emission Model

#### Dynamic degradation of the Yedoma Layer

*F*_*Dy*_ [kg year^−1^], the GHG emissions due to the dynamic degradation of the Yedoma layer, is defined as

1 $$ {F}_{Dy}={F}_{Dy, dir}+{F}_{Dy,\sec }. $$

*F*_*Dy*, *dir*_: GHG emissions due to the release of gases trapped in the frozen soil [kg year^−1^]

*F*_*Dy*, sec_: GHG emissions due to the decomposition of organic matter [kg year^−1^]

The first term in Eq. (1) corresponds to direct emissions, while the second term represents secondary emissions due to dynamic degradation. The direct emissions are formulated as follows:

2 $$ {F}_{Dy, dir}=\Delta  {V}_{Dy}\times {X}_{\mathrm{GHG}}. $$

*∆V*_*Dy*_: Volume of thawed permafrost due to dynamic degradation [m^3^ year^−1^]

*X*_*GHG*, *i*_: GHG mass in thawed permafrost [kg m^−3^]

Observational studies have measured the settling velocity of the ground surface due to permafrost thawing in the area where fire has occurred (e.g., Iwahana et al. 2016). In this study, the volume of dynamic permafrost thawing is formulated based on this observational knowledge as follows:

3 $$ \Delta  {V}_{Dy}={P}_{dstrb}\times {A}_{ydm}\times {V}_{dstrb}. $$

*P*_*dstrb*_: Probability of occurrence of fire

*A*_*ydm*_: Area of Yedoma layer in a 1-degree grid cell [m^2^]

*V*_*dstrb*_: Settling velocity of the ground due to permafrost thawing [m year^−1^]

Equation (2) describes the processes of permafrost thawing with land subsidence owing to the occurrence of fire. We determine the fire area in the Yedoma layer with the first and second terms (*P*_*dstrb*_ × *A*_*ydm*_) in Eq. (3). The probability of fire, *P*_*dstrb*_, is given as a function of meteorological data based on the observed relationship between past occurrences of fires and meteorological conditions. Veraverbeke et al. 2017 showed high correlations between fire occurrence and temperature, total precipitation and convective precipitation in the Northwest territory (NT) and Alaska (AK) from 2001 to 2015. In this study, the future fire area ratio, *P*_*dstrb*_, is estimated using future meteorological data and the relationship shown below:

4 $$ {P}_{dstrb}=a+b\times {T}_{air}+c\times {P}_{total}+d\times {P}_{conv} $$

where *T*_*air*_ is surface air temperature [K], *P*_total_ is total precipitation [kg/m^2^/s], and *P*_*conv*_ is convective precipitation [kg/m^2^/s], and the coefficients are *a* = − 0.495, *b* = 0.00179, *c* = − 343.6, *d* = 204.4. The coefficients in Eq. (4) are obtained using multiple regression of the fire area ratios for 2001-2015 in NT and AK, from Veraverbeke et al. 2017, and NCEP reanalysis data (Kalnay et al. 1996) for the same regions. To estimate the future fire area ratio, the bias of the global climate models (GCMs, details of which are explained later) is corrected with NCEP reanalysis data (Kalnay et al. 1996) by subtracting the climatological error (the difference between model results and the reanalysis data using 1980–2000 average). As a result of this bias correction, the estimated fire area ratio based on Eq. (4) is consistent with past observations (Veraverbeke et al. 2017). Given that Veraverbeke et al. 2017 found correlation based on the NT and AK regions, we estimate the future fire area ratio by averaging the climate model data at 10-degree resolution. We also confirmed that the difference between the estimated value of the fire area obtained by Eq. (4) and the observed value (Veraverbeke et al. 2017) has a normal distribution (not shown, with standard deviation = 0.00229). Considering that fires generally occur stochastically, a normal distribution with the above standard deviation, corresponding to the difference between the estimated and observed fire area ratio, was used to randomly assign values to each 1-degree grid.

With respect to the area of the Yedoma layer, *A*_*ydm*_, we use the results of Saito et al. (2020) regarding the behavior of soil moisture and organic carbon from the last interglacial period (approximately 120,000 years ago) to the present with 20 km resolution. Since the Yedoma layer is considered to be a region where soil frozen water and soil organic carbon are particularly concentrated (e.g., Strauss et al. 2017), in this study, we defined the Yedoma layer by using a threshold value for soil frozen water and soil organic carbon as calculated in Saito et al. 2020. We based our threshold value on the “vulnerability” measure defined in Saito et al. 2020 as (ICE/max(ICE) × SOC/max(SOC)), where ICE and SOC are soil frozen water and soil organic carbon, respectively, and “max” denotes the maximum value across the spatial dimension. According to Strauss et al. 2017, the soil organic carbon in the Yedoma layer is estimated to be 83–129 GtC. In this study, the threshold of vulnerability was chosen so that the soil organic matter of the Yedoma layer falls within the range of Strauss et al. 2017 (Table 1).

The settling velocity, *V*_*dstrb*_, in Eq. (2) is defined based on observational studies. Table 1 of Iwahana et al. 2016 synthesized the annual ground subsidence rates at various fire-burnt sites. In this study, the average value of Iwahana et al. 2016 is used; the range of the sedimentation velocity over the fire-bunt region is 2.4 ± 2.1 cm/year, as shown in Table 1.

The GHG concentration, *X*_*GHG*_, in Eq. (1) can be expressed by the following equation:

5 $$ {X}_{GHG}={R}_{pore}\times {C}_{GHG}\times {\rho}_{GHG}. $$

*R*_*pore*_: Volume fraction of bubbles in the permafrost [ratio]

*C*_*GHG*_: GHG concentration in the permafrost pores [ratio]

*ρ*_*GHG*_: Mass density of GHG [kg m^−3^]

In this study, we consider CO_2_ and CH_4_ as the GHG emissions and use data obtained by field observation in the Yedoma layer in Alaska and Siberia (Saito et al. 2017) to set the values of *R*_*pore*_ and *C*_*GHG*_. As reported in Saito et al. 2017, the *R*_*pore*_ and *C*_*GHG*_ values obtained by field observation have very large variation. Table 2 shows the standard deviation of the observed values in Saito et al. 2017. In calculating the dynamic degradation, the average value for the ground ice and frozen soil is used for the calculation of *X*_*GHG*_. It is reported that the ice content in the Yedoma layer (*r*_ice_) is approximately 0.64 (Strauss et al. 2017). Accordingly, the ratios of ground ice and frozen soil (*r*_ice_ and 1 − *r*_ice_, respectively) are used as multipliers for *R*_pore_ and *C*_GHG_. Field observations revealed that the layer with high GHG concentration (Table 2) was above (approximately) 5 m in the soil column and that the lower layer had very low GHG concentration. In this study, therefore, we assume that the GHG concentration, as shown in Table 2, is zero below 5 m.

**Table 2:** Volume fraction of air bubbles in the ground ice and frozen soil (*R*_pore_) and the concentration of GHGs in the air bubbles (*C*_GHG_)

Variable	CO_2_/ground ice	CH_4_/ground ice	CO_2_/frozen soil	CH_4_/frozen soil
*R*_pore_ [cc/cc]	0.044 (0.021)	0.031 (0.004)	0.019 (0.010)	0.014 (0.0074)
*C*_GHG_ [ppmv]	2992 (5101)	21848 (38434)	7714 (11933)	131675 (139746)

The average value and standard deviation for CO_2_ and CH_4_ are shown

In order to estimate the GHG emissions associated with the decomposition of soil organic carbon due to the dynamic degradation of permafrost (*F*_*Dy*, sec_ in Eq.1), this study considers four types of decomposition, following Schneider von Deimling et al. 2015 and Gasser et al. 2018. Specifically, we differentiate decomposition types based on two types of organic matter quality (fast or slow) and two types of soil moisture conditions (aerobic or anaerobic). The following equations for the decomposition of thawed permafrost carbon are solved with a global resolution of 1 degree:

6 $$ \frac{d{C}_{\mathrm{thaw}}^{i,j}}{dt}={\pi}^{i,j}{F}_{\mathrm{thaw}}-\frac{R^j}{\tau^i}{C}_{\mathrm{thaw}}^{i,j} $$

*i*: index for the quality of soil organic matter (fast or slow decomposition)

*j*: index for the soil moisture state (aerobic and anaerobic decomposition)

${C}_{\mathrm{thaw}}^{i,j}$: soil organic carbon content in the thawed permafrost [kg]

*F*_thaw_: flux of soil organic carbon due to permafrost thawing [kg/year]

*π*^*i*, *j*^: fraction of flux for the corresponding types

*τ*^*i*^: turnover time of soil organic carbon [year]

*R*^*j*^: changes in soil organic carbon decomposition rate due to temperature rise

The model parameter, *π*^*i*, *j*^, i.e., the fraction of thawed soil organic carbon, depends on the quality of organic matter (*i* = 1: fast, *i* = 2: slow decomposition) and soil water content (*j* = 1: aerobic, *j* = 2, anaerobic). The quality of organic matter is an important determinant for the timescale of the carbon release (Strauss et al. 2015). We subdivide the thawed permafrost carbon into a fast and slow decomposing fraction with annual and decadal timescales (*τ*^*i*^) based on the literature of soil organic quality, as shown in Table 1 (Sitch et al. 2003; Dutta et al. 2006; Koven et al. 2011; Burke et al. 2012; Schädel et al. 2014).

The soil water content is also a key determinant in the decomposition of soil organic carbon. In this study, the fraction of thawed permafrost carbon under the aerobic or anaerobic condition is determined by the wetland fraction, *r*_*wtlnd*_, obtained from the Global Lakes and Wetland Database (Lehner and Döll 2004). The original wetland fraction map is interpolated into 1-degree grid cells. The fraction of soil organic carbon for aerobic decomposition is 1 − *r*_*wtlnd*_, while that for anaerobic decomposition is *r*_*wtlnd*_ in each grid cell. In the future simulations, extensions of the wetland area are represented as a function of surface air temperature rise, with reference to Schneider von Deimling et al. 2015. Specifically, we describe the increase in *r*_*wtlnd*_ by linear scaling with the surface air temperature anomaly, *∆ T*_*a*_ (the anomaly is calculated as the difference from the first 20-years average). The wetland fraction reaches its maximum extent (*∆r*_*wtlnd*, max_) for a warming *∆ T*_*a*_ of 10 K. For further warming, the wetland fraction is kept constant at the maximum extent. The uncertainty range of *∆r*_*wtlnd*, max_ is shown in Table 1.

The flux of soil organic carbon due to permafrost thawing is formulated as

7 $$ {F}_{\mathrm{thaw}}=\Delta  {V}_{Dy}\times {\rho}_{SOC} $$

Here, *ρ*_*SOC*_ is the density of soil organic carbon, calculated as ${\rho}_{SOC}=\frac{\sigma_{SOC}}{d_{SOC}}$, where *σ*_*SOC*_ is the soil organic carbon from Saito et al. 2020, and *d*_*SOC*_ is the depth of the soil organic carbon. *d*_*SOC*_ is a model parameter in the range shown in Table 1. The changes in soil organic carbon decomposition rate due to temperature change are formulated with reference to Schneider von Deimling et al. 2015 as follows:

8 $$ {R}^j=Q{10}^{j\ \left({T}_g-10\right)/10} $$

*Q*10^*j*^: temperature sensitivity parameter

*T*_*g*_: soil temperature [°*C*]

*Q*10^*j*^ is the temperature sensitivity of carbon decomposition due to the microbial soil activity rises that accompany increasing soil temperature. The *Q*10^*j*^ parameter is dependent on the aerobic or anaerobic conditions; the parameter ranges are given based on the literature, as shown in Table 1 (Walter and Heimann 2000; Shadel et al. 2013; Schneider von Deimling et al. 2015). For *T*_*g*_, we use monthly mean soil temperature (averaged over the top 4 m), calculated by land-surface model simulations with 1-degree resolution (Yokohata et al. 2020a, 2020b). The details of this are explained in Section 2.2.

The GHG emissions due to the decomposition of soil organic carbon, *F*_*Dy*, *sec*_, can be calculated by solving ${C}_{\mathrm{thaw}}^{i,j}$ in Eq. (5) as follows:

9 $$ {F}_{Dy,\sec }={\sum}_{i,j}\left(\frac{d{C}_{\mathrm{thaw}}^{i,j}}{dt}-{\pi}^{i,j}{F}_{\mathrm{thaw}}\right)\times {r}_{\mathrm{gas}}^{i,j}\left(1-{oxd}^j\right) $$

${r}_{gas}^{i,j}$: production ratio of GHG (CO_2_ or CH_4_) due to soil organic matter decomposition

*oxd*^*j*^: oxidation rate of CH_4_

The production rate of CO_2_ and CH_4_, ${r}_{\mathrm{gas}}^{i,j}$, is dependent on the soil organic quality and aerobic or anaerobic conditions. The ranges of the parameter values for ${r}_{\mathrm{gas}}^{i,j}$ are determined based on incubation studies under various conditions (Segers 1998; Schuur et al. 2008; Lee et al. 2012; Walter Anthony et al. 2014; Schneider von Deimling et al. 2015). The *oxd*^*j*^ term corresponds to the fraction of released carbon that is oxidized (thus, *oxd*^*j*^ = 0 for CO_2_), the range of which is determined from the literature (Burke et al. 2012; Schneider von Deimling et al. 2015).

#### Thermodynamic degradation of the permafrost layer

In this section, the thermodynamic degradation of the permafrost (i.e., the thickening of the active layer) due to the rise in soil temperature in future climate change is formulated. In the formulation for the dynamic degradation of the Yedoma layer (Eq.1), direct emissions are considered due to the presence of high-concentration GHGs (Table 1). However, direct emissions are not considered in the thermodynamic degradation since high-concentration GHGs are not expected to be present in the permafrost other than in the Yedoma layer. Even in the thermodynamic degradation, (Eqs. 5, 6, 7 and 8) is used for the formulation of the secondary GHG release. To establish the flux of soil organic carbon due to the permafrost thawing associated with thermodynamic degradation, Eq. (6) is replaced by

10 $$ {F}_{\mathrm{thaw}}=\Delta  {V}_{thr}\times {\rho}_{SOC} $$

*∆V*_*Dy*_: Volume of thawed permafrost due to thermodynamic degradation [m^3^ year^−1^]

Here, we use the same *ρ*_*SOC*_ as described in Eq. (6). For the volume of thawed permafrost due to thermodynamic degradation, numerical simulations using a global land-surface model (Yokohata et al. 2020a, 2020b) are used. The formulation for *∆V*_*Th*_ [m^3^ yr^-1^] in the *t*th year is as follows:

11 $$ \Delta  {V}_{Th}=\left[ ALT(t)-\operatorname{MAX}\left( ALT\left({t}_0\right),\kern0.5em {t}_0=0,\dots t-1\right)\right]\times {A}_{grid}. $$

Here, *ALT*(*t*) is the active layer thickness [m] (ALT, the annual maximum thaw depth) in the *t*th year. The active layer is defined as the region where the ground temperature exceeds 0 °C in summer seasons. Eq. (10) is formulated in order to avoid counting the thawed region multiple times due to the annual variability of ALT. *A*_grid_ in Eq. (10) is the grid area of the global climate model used for the simulation (1-degree latitude and longitude) as described in the next section. If Eq. (10) produces a negative value, *∆V*_*Th*_ is set to zero.

### Experimental setting

Table 2 shows the standard values and uncertainty ranges for all parameters given in this study, as explained in the previous sections. Each parameter was randomly selected from the uniform distribution with the uncertainty range shown in Table 2. In all, 500 simulations were performed using the randomly selected parameters.

In addition to the parameters shown in Table 2, one of the physical variables used in the Permafrost Degradation and Greenhouse gasses Emission Model (PDGEM) model is the soil temperature, *T*_*g*_, which is used for changes in the soil decomposition rate (Eq. 7) and the volume of the thermodynamic permafrost thawing (Eq. 9). *T*_*g*_ is calculated by the global land-surface climate model MIROC-INTEG-LAND (MIROC INTEGrated LAND surface model, Yokohata et al. 2020a, 2020b), which is based on the land surface components of the global climate model MIROC (Model for Interdisciplinary Research on Climate; Watanabe et al. 2010; Takata et al. 2003). The results of multi-GCM simulations provided by the Inter-Sectoral Impact Model Inter-comparison Project phase 1 (ISIMIP1, Hempel et al. 2013) were used as the atmospheric forcings to drive this land surface model. Using atmospheric forcings generated by the five GCMs (GFDL-ES2M, Dunne et al. 2012; HadGEM2-ES, Jones et al. 2011; IPSL-CM5A-LR, Dufresne et al. 2013; Nor-ESM, Bentsen et al. 2013; MIROC-ESM-CHEM, Watanabe et al. 2011) of ISIMIP1, we performed historical simulations (1951–2005) and future simulations (2006–2100) based on representative concentration pathways (RCP, van Vuuren et al. 2011) RCP2.6 and RCP8.5. The resolution of the land surface model was 1 degree (Nitta et al. 2014). In this study, a model version with improved permafrost processes (Yokohata et al. 2020b) was used.

Another physical variable given to the PDGEM model was the future temperature change, *∆T*_*a*_, which is used for the future extent of the wetland area. We use the future projections of the five ISIMIP1 GCMs (Hempel et al. 2013) noted above, under the RCP2.6 and RCP8.5 scenarios for *∆T*_*a*_.

The GHG emissions due to the dynamic and thermodynamic permafrost thawing are calculated with the model parameters shown in Table 2. The GHG emissions are then integrated globally and given to a simplified climate model, ACC2 (Tanaka and O’Neill 2018), which calculates the global mean surface air temperature response to GHG emissions. By calculating the global mean surface air temperature response with and without the permafrost GHG emissions under RCP 8.5, the impact of permafrost thawing on the climate system can be examined.

## Results and discussion

**Fig. 1:**
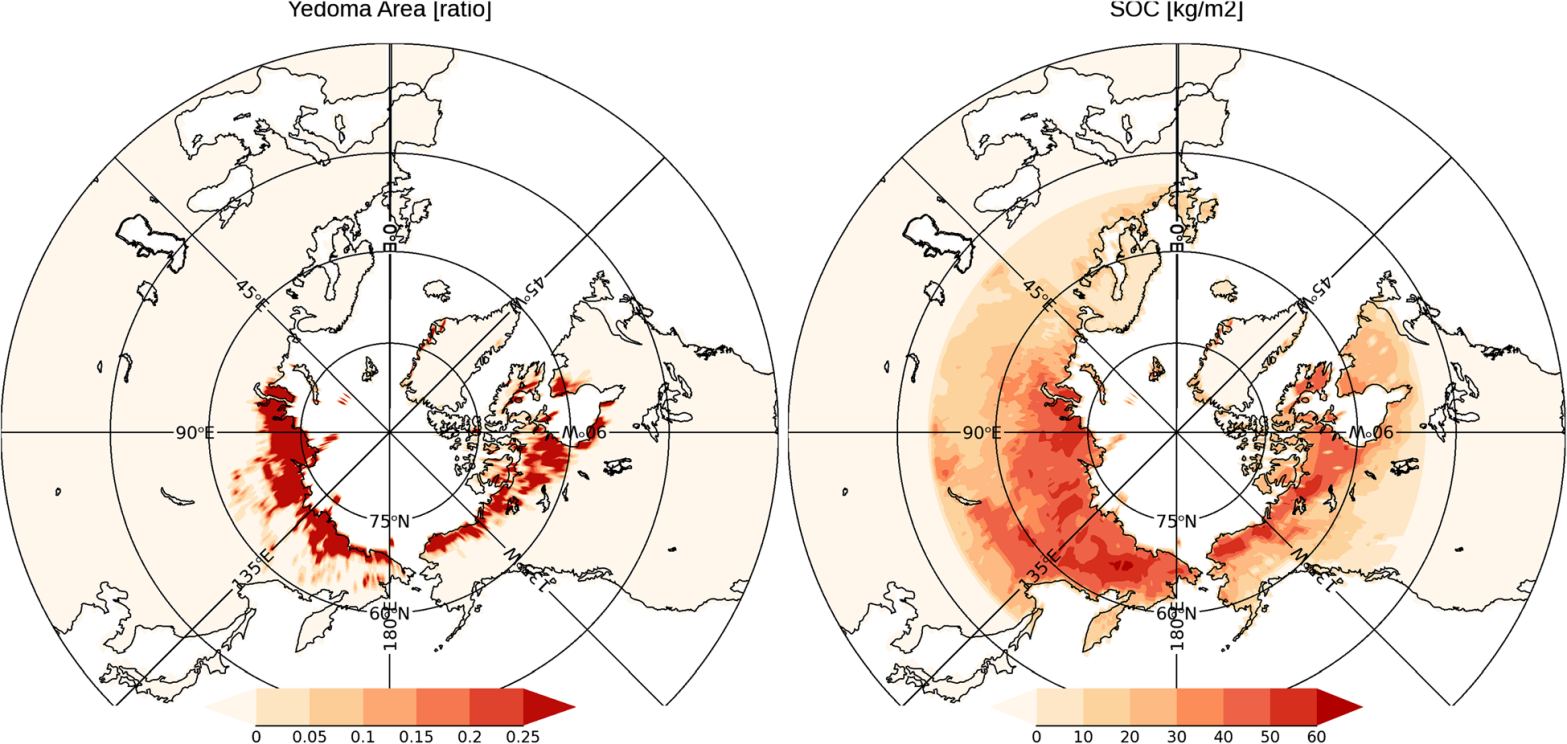
(Left) Yedoma area used in the model simulation (unit = ratio to grid area); (right) soil organic carbon used in the model simulation (unit = kg/m^2^)

Figure 1 shows the area ratio of the Yedoma layer and the distribution of soil organic carbon used to calculate the dynamic degradation. As described in the previous section, this study defines the Yedoma layer as permafrost having a particular abundance of soil organic carbon and soil frozen water, based on data from Saito et al. (2020). The total soil organic carbon in the Yedoma layer, as shown in Fig. 1, is consistent with the estimates of Strauss et al. 2017 (106 GtC, the middle of uncertainty range 83-129 GtC). Soil organic carbon in the Arctic has accumulated in cold and humid environments where soil degradation is slow. It is distributed in eastern Siberia and Alaska, found mostly in coastal areas and near river basins (Saito et al. 2020). These areas are characterized by extremely low temperatures (Yokohata et al. 2020b).

Table 3 shows the cumulative emissions of CO_2_ and CH_4_ due to the dynamic and thermodynamic degradation of the permafrost in the RCP8.5 scenario. Before conducting our future experiments, we confirmed that the average value of CH_4_ emission (3.9 TgCH_4_) for the 5-year period from the start year (2006) of the calculation is close to the estimate of present CH_4_ emission (~ 4 TgCH_4_, Walter Anthony et al. 2016; ~ 1 TgCH_4_, Saunois et al. 2020). As indicated in Table 3, the CO_2_ and CH_4_ releases due to the dynamic degradation (direct plus secondary emissions) of the Yedoma layer are approximately 0.1 PgC and 5 TgCH4, respectively. In each case, this is less than 1% of the total release due to dynamic and thermodynamic degradation (47 PgCO2 and 2067 TgCH4, respectively). Comparing the direct release of GHGs trapped in the ground ice and frozen soil and the secondary release of GHG due to the decomposition of soil organic carbon, the latter is an order of magnitude larger than the former (Table 3). Even though very high concentrations of CO_2_ and CH_4_ are contained in the ground ice and frozen soil of the Yedoma layer (Saito et al. 2017), their impact on the climate is quite small when they are released into the atmosphere by the degradation of the permafrost. In the present formulation and over the study period (up to 2100), the dynamic degradation of the Yedoma layer does not significantly affect the carbon cycle feedback.

**Table 3:** Future predictions of GHG emissions from permafrost degradation in the RCP8.5 scenario

	CO2 emissions [TgC or PgC]	CH4 emissions [GgCH_4_ or TgCH_4_]	Global mean temperature change [°C]	Reference
Dynamic, Direct	3 (1–5) TgC	123 (43–203) TgCH_4_	–	This study
Dynamic, Direct + secondary	0.1 (0.04–0.2) PgC	5 (2–8) TgCH_4_		
Total: Dynamic + Thermo-dynamic	47 (31–63) PgC	2067 (1261–2821) TgCH_4_	0.08 (0.05–0.11)	This study
	48 (32–66) PgC			
Total: previous studies	87 (42–141) PgC	1474 (836–2614) TgCH_4_	0.09 (0.05–0.14)	a
	226 PgC			b
	27 (− 41 to 95) PgC			c
	59 (11–143) PgC			d
	57 (28–113) PgC			e
	37–174 PgC			f
	37–343 PgC		0.29 (0.17–0.41)	g

The cumulative CO_2_ and CH_4_ emissions are estimated at the end of twenty-first century. Direct emissions due to dynamic degradation, direct plus secondary emissions due to dynamic degradation, and total emissions due to dynamic plus thermodynamic degradation in the current study are shown (average value and 68th percentile range of model ensemble simulations). Some estimated values reported in previous studies are also shown in the far right column. References for the estimate in the previous studies are as follows. a: Schneider von Deimling et al. (2015), b: MacDougall et al. (2015), c: McGuire et al. (2018), d: Gasser et al. (2018), e: Koven et al. (2015), f: Schuur et al. (2015), g: Schaefer et al. (2014)

As shown in Table 3, the cumulative CO_2_ and CH_4_ emissions (the emissions due to dynamic and thermodynamic degradation) in the RCP8.5 scenario estimated in the present study are 47 PgC (31–63 PgC, 68% range) and 2067 (1261–2821) TgCH4, respectively. For comparison, Table 3 also shows the amount of GHG gas emissions estimated in various previous studies. As can be seen in the table, these estimated emissions cover a wide range. Notably, the GHG emissions for the RCP8.5 scenario estimated in the present study are within the indicated range of uncertainty. As shown in the table, the aggregated carbon content of CO_2_ plus CH_4_ emissions due to permafrost degradation in the present study is 48 (32–66) PgC, and the increase in surface air temperature due to permafrost degradation is 0.08 (0.05–0.11) °C. Other studies (e.g., Schaefer et al. 2014; Schneider von Deimling et al. 2015; Koven et al. 2015; Gasser et al. 2018) have reported similar values. One multi-model study featuring state-of-the-art process models reported that in some of the models, atmospheric carbon may actually be absorbed due to permafrost degradation owing to the effect of potential plant growth after thawing (McGuire et al. 2018). The spread in estimated GHG emissions in McGuire et al. 2018 is larger than in other studies, ranging from a carbon sink of 41 PgC to a carbon source of 140 PgC at the end of the twenty-first century**.** On the other hand, the amount of CH4 released in the RCP8.5 scenario in the present study is larger than the 1474 TgCH_4_ reported by Schneider von Deimling et al. (2015).

Table 4 shows estimates of GHG emissions in the RCP2.6 scenario. Even here, the dynamic degradation of the Yedoma layer contributes less than 1% to total GHG emissions, and the direct release of dynamic degradation is an order of magnitude smaller than the secondary release. As in the RCP8.5 scenario, the cumulative emissions of CO_2_ and CH_4_ resulting from the combined effect of dynamic and thermodynamic degradation are similar to those in previous studies. In the present study, the combined carbon content of CO_2_ and CH_4_ emissions is 22 (15–29) PgC, which is similar to the total 27 PgC, reported by Gasser et al. (2018). On the other hand, the amount of released CH_4_ is 986 (618–1341) TgCH_4_, which is larger than the 446 TgCH4 estimated in Schneider von Deimling et al. (2015). The increase in surface air temperature due to permafrost degradation is 0.05 (0.03–0.07) °C, which is similar to the 0.06 (0.03–0.10) °C estimated in Schneider von Deimling et al. (2015).

**Table 4:** Same as Table 3, but for the RCP2.6 scenario

	CO2 emissions [TgC or PgC]	CH4 emissions [GgCH_4_ or TgCH_4_]	Global mean temperature change [°C]	Reference
Dynamic, Direct	1 (0.4–2) TgC	48 (17–81) GgCH4	–	This study
Dynamic, Direct + secondary	0.07 (0.03–0.1) PgC	3 (1–5) TgCH4		
Total: Dynamic + Thermo-dynamic	21 (14–28) PgC	986 (618–1341) TgCH_4_	0.05 (0.03–0.07)	This study
	22 (15–29) PgC			
Total: Previous studies	36 (20–58) PgC	446 (218–921) TgCH4	0.06 (0.03–0.10)	a
	103 PgC			b
	27 (6–62) PgC			d
	56 (13–118) PgC			h

References for the estimate in the previous studies are as follows. a: Schneider von Deimling et al. (2015), b: MacDougall et al. (2015), d: Gasser et al. (2018), h: MacDougall and Knutti (2016)

**Fig. 2:**
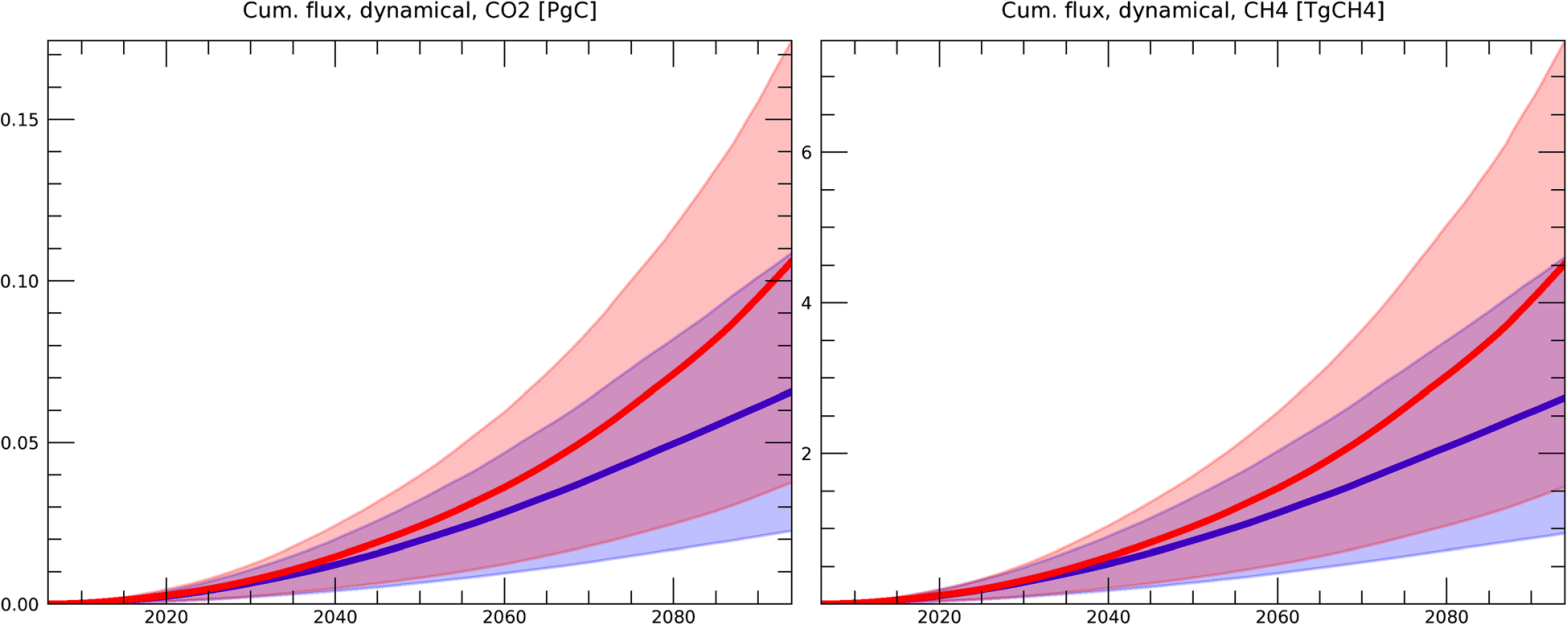
Cumulative GHG flux due to dynamic degradation of the Yedoma layer for CO_2_ (left, unit = PgC) and CH_4_ (right, unit = TgCH4). The simulations under the RCP2.6 (blue) and RCP8.5 (red) scenarios are shown. The width of the colored area represents the 68 percentiles of the simulated results forced by five GCMs, each of which involved 300 simulations with different model parameters sampled from the uncertainty ranges shown in Table 1. The average value of the model simulations is represented by the bold blue (RCP2.6) and red (RCP8.5) lines

**Fig. 3:**
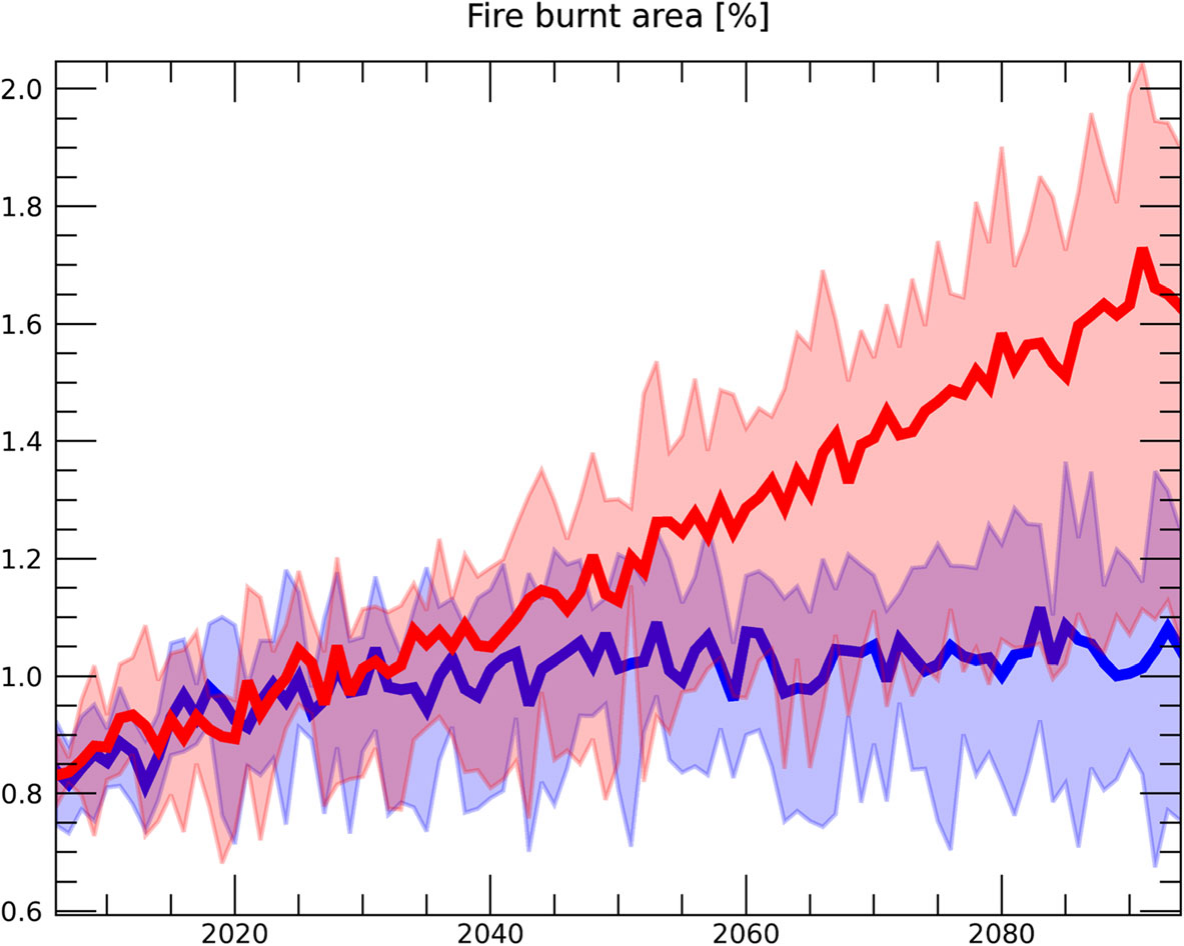
Time sequence for the probability of fire in the RCP8.5 (red) and RCP2.6 (blue) scenarios. Unit is the ratio (%) to the total land area above 50° N. The width of the colored area represents the 68 percentiles of the 300 simulations with different model parameters, as explained in Section 2.1.1. The average value of the model simulations is represented by the bold blue (RCP2.6) and red (RCP8.5) lines

Figure 2 shows the cumulative GHG release due to dynamic permafrost degradation. In the formulation of dynamic degradation, GHG emissions are dependent on the possibility of fire (*P*_*dstrb*_ in Eq. 4) and the subsidence velocity of the land surface (*V*_*dstrb*_), both of which are based on present observation (Section 2.1.1). Since we use the same *V*_*dstrb*_ for the RCP8.5 and RCP2.6 scenarios, the difference between the scenarios in Fig. 2 can be attributed to the difference in *P*_*dstrb*_. In our study, the possibility of fire is increased mainly due to temperature rise, as shown in Fig. 3, since *P*_*dstrb*_ is estimated as a function of meteorological data (Eq. 4) based on the relationship established from historical data (Veraverbeke et al. 2017).

**Fig. 4:**
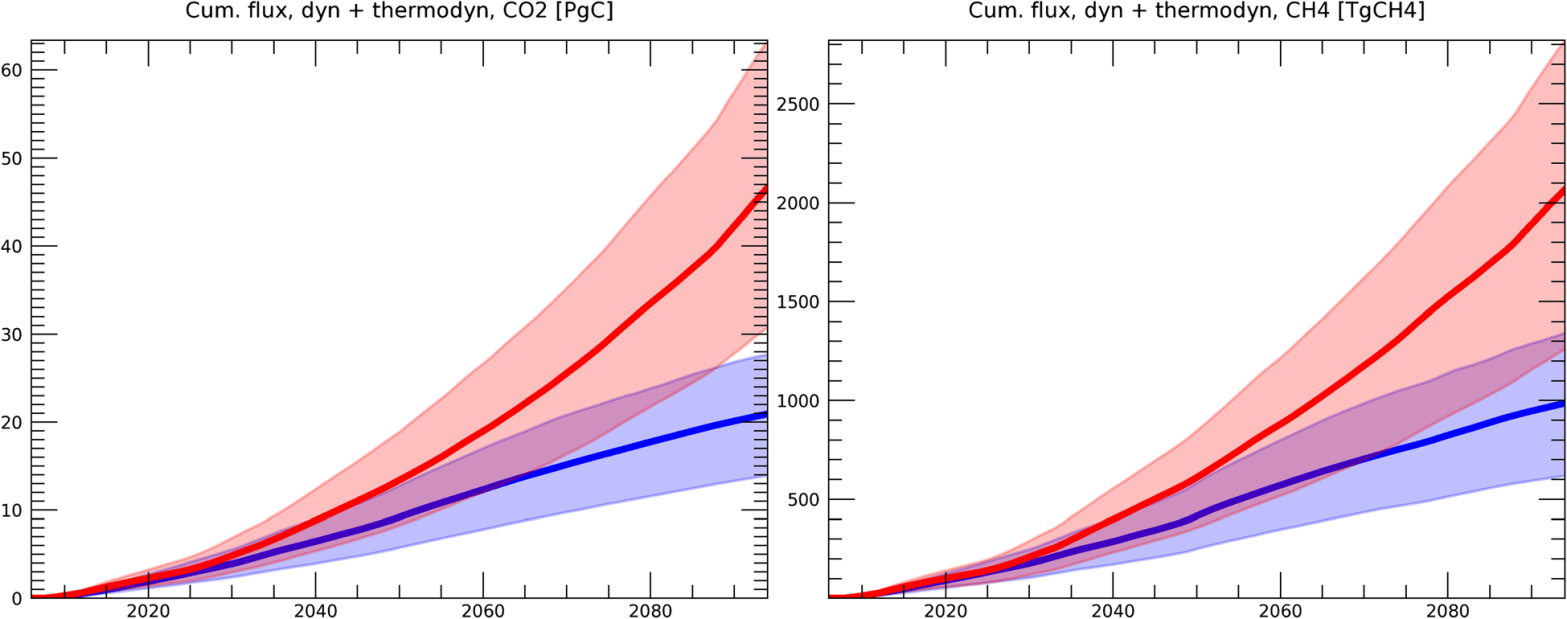
Same as Fig. 2 but for the cumulative GHG flux due to the total (dynamic plus thermodynamic) degradation of permafrost for CO_2_ and CH_4_, respectively

Figure 4 shows the results of the cumulative release of GHG from the combination of dynamic and thermodynamic degradation. As described above, since the contribution of dynamic degradation of the Yedoma layer is less than 1% of the total, the cumulative emission is essentially determined by thermodynamic degradation (Section 2.1.2). This thermodynamic degradation is obtained by solving the equation of secondary release shown in Eq. (8), based on Eqs. (9)–(10). Here, the change in active layer thickness (ALT) simulated by the global land surface model (Yokohata et al. 2020a, 2020b) is used for the calculation of permafrost degradation. As shown in Fig. 4, the cumulative release of CO_2_ from permafrost degradation increases almost linearly in RCP2.6, but the rate of increase rises in RCP8.5 in the latter half of the twenty-first century. This is due to the fact that the permafrost area rapidly decreases in RCP8.5 in the latter half of the century in these simulations (the details of the land surface model simulation results are provided in Yokohata et al. 2020b).

**Fig. 5:**
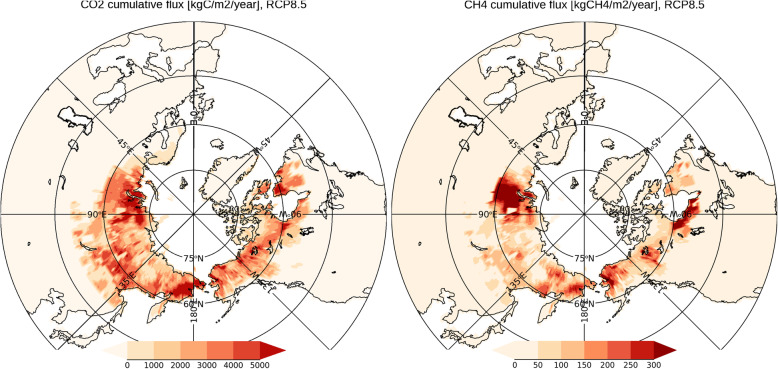
The cumulative CO_2_ flux [TgC] (bottom, left) and CH_4_ flux [TgCH4] (bottom, right) due to the permafrost degradation (dynamic + thermodynamic) at the end of the twenty-first century in the RCP8.5 scenario. The average of all ensemble members is shown

**Fig. 6:**
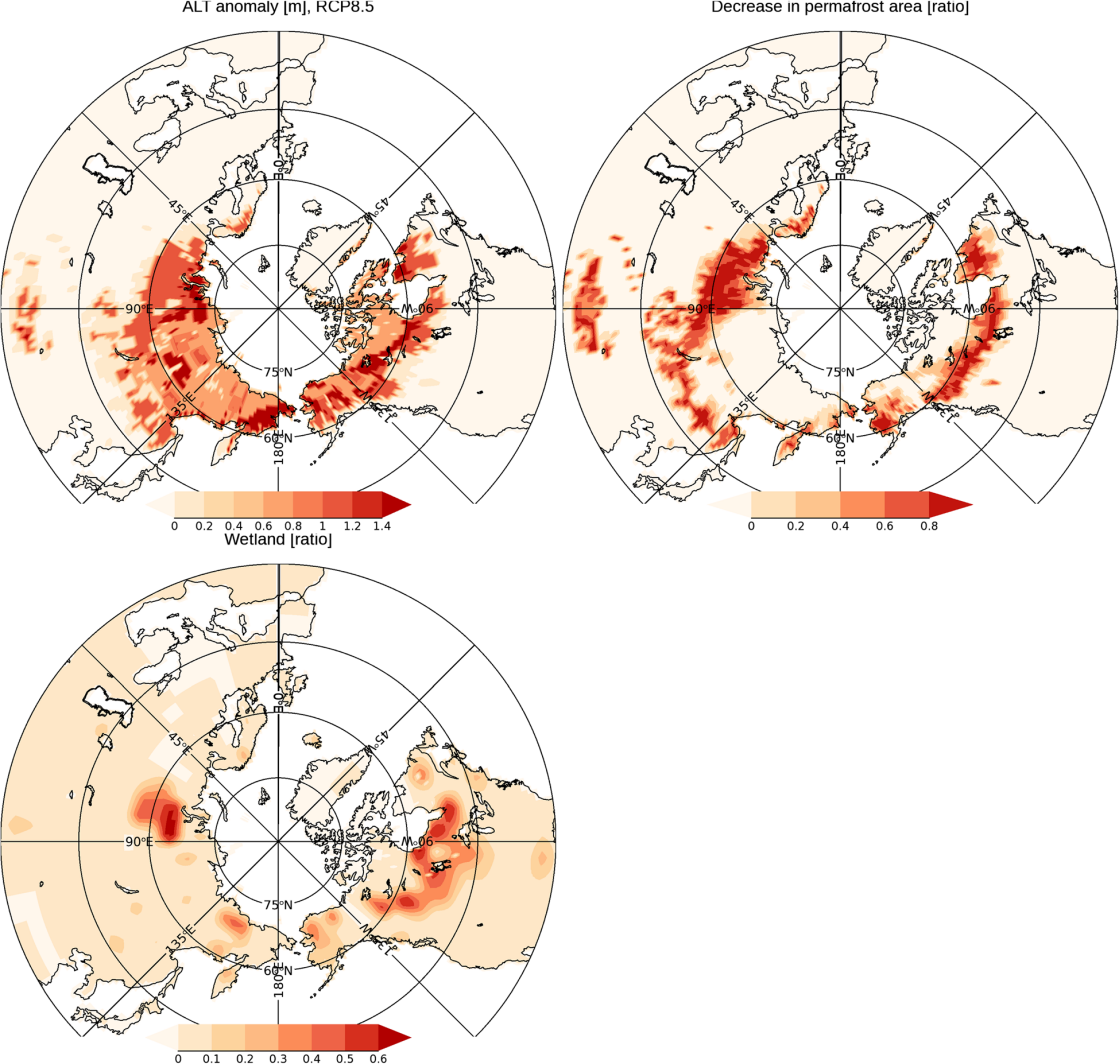
Total change in active layer thickness [m] (top left), decrease in the permafrost area (top right), the wetland fraction (bottom) at the end of the twenty-first century in the RCP8.5 scenario. The average of all ensemble members is shown

Figure 5 shows the CO_2_ and CH_4_ emissions at the end of the twenty-first century in the RCP8.5 scenario. We found that CO_2_ emissions are more widespread compared to the confined emissions of CH_4_. This is related to the fact that CH_4_ emissions can be larger in a wetland region, and the regions with a high wetland ratio are limited. The important factors that determine thermodynamic degradation are changes in the active layer thickness (Eq. 10) and the rise of soil temperature (Eq. 7). In order to interpret the results in Fig. 5, the changes in the active layer thickness, permafrost area, and wetland fraction are shown in Fig. 6. As indicated in the figure, the changes in active layer thickness are large in western and eastern Siberia, and in the North America coastal regions of the Arctic Ocean. This distribution roughly corresponds to that of CO_2_ emissions (Fig. 5). In western and eastern Siberia, and the northern part of North America, the amount of CH_4_ emission is large in regions with a large wetland fraction (Fig. 6).

Figure 6 also shows the changes in the permafrost area, which corresponds to the region with temperatures below 0 °C throughout the year. In the regions where the permafrost area decreased, the area below 0 °C throughout the year decreased. Figure 6 indicates that the permafrost area decreases significantly in the western and southern part of eastern Siberia, while permafrost remains in a wide region from the center to eastern Siberia. In other words, at the end of the twenty-first century, permafrost will remain in the cold regions, with the expectation that thawing will progress in the twenty-second century. Previous studies have reported that the impact of permafrost degradation on the climate will be greater after the end of the twenty-first century (e.g., McGuire et al. 2018), which is consistent with our result.

## Conclusions

In this study, we developed PDGEM, a model for estimating GHG emissions due to permafrost degradation. Using the model, we produced future projections of the following three processes:

- Direct release of GHGs due to the dynamic degradation of the Yedoma layer: The process in which high concentrations of CO_2_ and CH_4_ trapped in the ground ice and frozen soil of the Yedoma layer are released due to dynamic degradation.
- Secondary release of GHGs due to the dynamic degradation of the Yedoma layer: The process by which organic matter trapped in the Yedoma layer is newly decomposed by the thawing of the permafrost to release CO_2_ and CH_4_.
- Secondary release of GHGs due to the thermodynamic degradation of permafrost: The process by which organic matter trapped in the permafrost is newly decomposed by the thawing of the permafrost to release CO_2_ and CH_4_.

In the RCP8.5 and RCP2.6 scenarios, numerical simulations through the twenty-first century showed that the combination of (a) plus (b) contributed less than 1% of the total emissions resulting from (a) + (b) + (c). It was also found that the contribution of (a) is an order of magnitude smaller than that of (b). The cumulative release of CO_2_ plus CH_4_ produced by (a) + (b) + (c) was 48 (32–66) PgC for RCP8.5, and 22 (15–29) PgC for RCP2.6. This is consistent with a recent multi-model study (− 41 to 95 GtC, McGuire et al. 2018) which reported that in one of the ESMs, the land becomes a carbon sink owing to the effect of plant growth after thawing.

In this study, dynamic degradation of the Yedoma layer (defined as the location of high soil organic carbon and soil frozen water) is formulated by the possibility of fire (*P*_*dstrb*_) and the present land surface subsidence velocity (*V*_*dstrb*_) as shown in Eq. (3). The contribution of dynamic degradation ((a) + (b) above) is small since the area ratio of the Yedoma layer (*A*_*ydm*_) is very small. The contribution of dynamic degradation will be large if the dynamic degradation (i.e., the subsidence of surface due to dynamic collapse) occurs outside the Yedoma layer, or if the subsidence velocity is higher than it is currently. To estimate the probability of fire, the relationship between the occurrence of fire and meteorological conditions (Eq. 4) constructed from observation data is used; however, if the relationship described in Eq. (4) is different in the future, the frequency of fires will also change.

With PDGEM, the global distribution of GHG emissions can be estimated (e.g., Fig. 5) by using the thawing process of permafrost obtained from a state-of-the-art land surface model (Yokohata et al. 2020a, 2020b), taking into account the substantial uncertainties associated with the model’s parameters (Table 1) and future atmospheric changes. This represents a significant advantage when compared to previous related studies (e.g., Schneider von Deimling et al. 2015; Gasser et al. 2018; McGuire et al. 2018). The models of permafrost degradation in previous studies were unable to predict the geographic distribution of GHG emissions due to their simplification of physical processes (Schneider von Deimling et al. 2015; Gasser et al. 2018). On the other hand, for state-of-the-art earth system models that incorporate advanced physical and carbon cycle processes (McGuire et al. 2018), it is difficult to fully consider the uncertainties in model prediction such as the uncertainties in future atmospheric responses. In this study, combining a simple scheme of carbon cycle processes with the results of the latest land surface model makes it possible to project the geographical distribution of future GHG emissions due to permafrost degradation (Fig. 5) by considering, across a very broad range, the uncertainties associated with the various model parameters and future atmospheric responses.

In the previous studies (e.g., Gasser et al. 2018), it has been shown that GHG emissions caused by the thawing of permafrost can be an obstacle to achieving the climate stabilization called for in the Paris Agreement. In addition, as described in Fig. 6, substantial permafrost remains unthawed at the end of the twenty-first century, and thus the impact of GHG gas emissions from permafrost thawing on the climate system is expected to increase markedly after that time (McGuire et al. 2018). As discussed in Section 4, the geographical distributions of GHG emissions (Fig. 5) are connected to changes in ground temperature, soil moisture status and wetland distribution, and the soil carbon accumulated over time scales of past glacial cycles. The hotspots with particularly large GHG emissions shown in Fig. 5 are determined by the interactions between these factors investigated in this study. In the regions of GHG emission hotspots shown in Fig. 5, it may be possible to reduce GHG emissions by taking measures such as restricting land development.

## Data Availability

Data sharing is not applicable to this article as no datasets were generated or analyzed during the current study. Please contact the authors for data requests.

## Abbreviations

ALT: Active layer thickness
CLAM: Circumpolar active layer monitoring
CMIP: Coupled Model Inter-comparison Project
GCM: Global climate model
GHGs: Greenhouse gases
IPA: International Permafrost Association
ISIMIP: Inter-Sectoral Impact Model Inter-comparison Project
MATSIRO: Minimal advanced treatments of surface interaction and runoff
MIROC: Model for Interdisciplinary Research on Climate
PDGEM: Permafrost Degradation and Greenhouse gases Emission Model
RCP: Representative concentration pathways
